# Overview of the Important Factors Influencing the Performance of Eco-Friendly Brake Pads

**DOI:** 10.3390/polym14061180

**Published:** 2022-03-16

**Authors:** Agustinus Purna Irawan, Deni Fajar Fitriyana, Cionita Tezara, Januar Parlaungan Siregar, Dwinita Laksmidewi, Gregorius Dimas Baskara, Mohd Zulkfly Abdullah, Ramli Junid, Agung Efriyo Hadi, Mohammad Hazim Mohamad Hamdan, Najid Najid

**Affiliations:** 1 Faculty of Engineering, Universitas Tarumanagara, Jakarta Barat 11440, Indonesia; najid@ft.untar.ac.id; 2 Department of Mechanical Engineering, Faculty of Engineering, Universitas Negeri Semarang, Kampus Sekaran, Semarang 50229, Indonesia; 3 Faculty of Engineering and Quantity Surveying, INTI International University, Nilai 71800, Negeri Sembilan, Malaysia; 4 College of Engineering, Universiti Malaysia Pahang, Gambang 26300, Pahang, Malaysia; ramli@ump.edu.my; 5 Faculty of Economics and Business, Universitas Katolik Indonesia Atma Jaya, Jl. Jenderal Sudirman No. 51, Jakarta 12930, Indonesia; dwinita.laksmi@atmajaya.ac.id; 6 School of Electrical Engineering and Informatics, Institut Teknologi Bandung, Bandung 40132, Indonesia; 13519190@std.stei.itb.ac.id; 7 School of Mechanical Engineering, Engineering Campus, Universiti Sains Malaysia, Nibong Tebal 14300, Penang, Malaysia; mezul@usm.my; 8 Mechanical Engineering Department, Faculty of Engineering, Universitas Malahayati, Jl. Pramuka No. 27, Kemiling, Bandar Lampung 35153, Indonesia; efriyo@malahayati.ac.id; 9 Faculty of Engineering and Computing, First City University College, No. 1, Persiaran Bukit Utama, Bandar Utama, Petaling Jaya 47600, Selangor Darul Ehsan, Malaysia; hazim.hamdan@firstcity.edu.my

**Keywords:** brake pad, natural fiber, filler, wear test, friction, composites

## Abstract

The braking system is a crucial element in automotive safety. In order for the braking mechanism to function effectively, the brake pads’ durability as well as quality are crucial aspects to take into account. A brake pad is a part of a vehicle that holds the wheel rotation so that braking can occur. Asbestos, which is harmful to human health, is a raw material that is recently being widely used as a material mixture for the manufacturing of brake pads. Many efforts have been made by researchers to find other natural alternative materials to replace the use of asbestos. Natural materials that have received much attention and research include coconut fiber, wood powder or flour, bamboo fiber, shell powder, etc. This review paper focuses on analyzing the main parameters that affect brake pad performance. The composition of filler and fiber types of reinforcement for polymer composites is discussed. Previous studies’ information on the fabrication and testing of brake pads are also highlighted. Furthermore, the findings of this review can provide researchers and academicians with useful information and points to consider for further research.

## 1. Introduction

Natural fibers have many advantages compared to synthetic fibers due to their low density, abundance, inexpensive, recyclable, biodegradable, renewable, and relatively high strength and stiffness characteristics [1]. Apart from their low cost and light weight, natural fiber reinforced composites are getting more attention because they are renewable, low energy consumption, and biodegradable. Aside from these benefits, increasing environmental concerns and rising petroleum consumption levels are driving the entire world to use more sustainable natural resources such as natural fibers [2,3,4,5]. Therefore, natural fibers are rising as reinforcing fibers for composite materials in the automotive, furniture, packaging, and construction industries due to these advantages [6].

**Figure 1:**
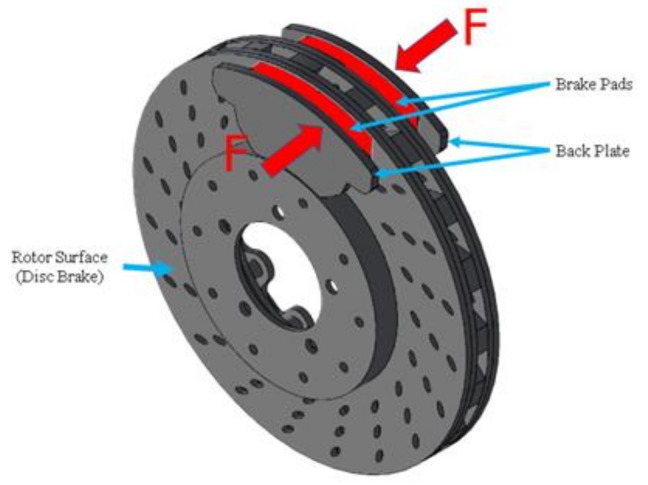
Force of friction between the brake pads and the rotor surface.

Asbestos is hydrated magnesium silicate with good characteristics, including strength, durability, flexibility, and resistance to corrosion, heat, and fire. Since it is plentiful, asbestos is a low-cost material. Its adaptability has resulted in its inclusion in various goods across various industries, including brake pads [7]. Brake pads are a part of the braking system components in addition to the master cylinder, wheel cylinder, and hydraulic control system. Brake pads have sparked a lot of research interest because of their nature and environmental impact. Binders, friction modifiers, fillers, and reinforcement are the four categories of materials utilized for brake pads production. Note that asbestos fibers are typically found in the polymeric matrix of brake pads, along with a variety of other compounds [8,9,10]. As shown in Figure 1, kinetic energy is converted into heat energy during the brake system operation by friction between the rotor surface and the brake pads [11,12,13].

**Figure 2:**
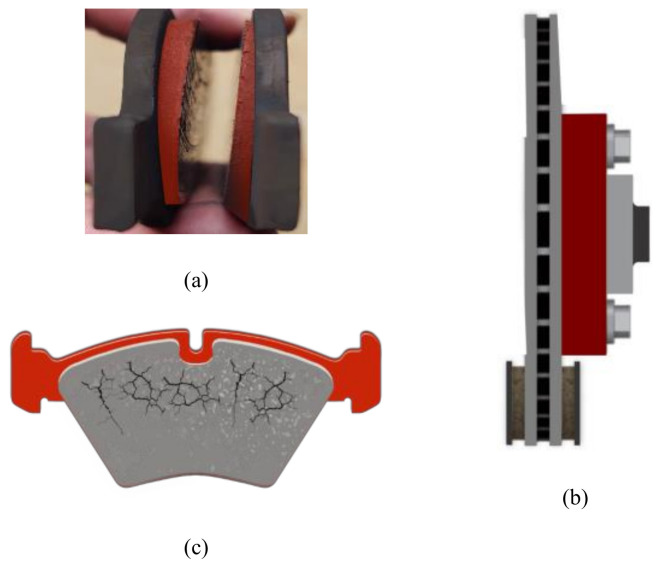
Failure on brake pads caused by excessive thermal loading (**a**) wears, (**b**) disc thickness variations, and (**c**) surface cracking.

Conduction transfers the heat energy created to the components in contact. As seen in Figure 2a–c, excessive thermal loading can cause judder (disc thickness changes), surface cracking, and severe wear on the contact surfaces. In addition, high temperatures can also cause brake fluid, seals, and other components to overheat, resulting in malfunctions in the braking system [11,12,13]. A brake absorbs energy with the help of friction force. As a result, when developing brake pad material, it must satisfy the following criteria: In diverse operating situations, for instance, temperature, pressure, speed, and environmental sustainability, decreased wear rates and a stable friction coefficient are achieved. This can be fulfilled with the right combination of materials used [8,11].

Based on the International Agency for Research on Cancer (IARC) and World Health Organization (WHO), asbestos is carcinogenic. It can cause cancer in the lungs from the dust produced by these materials. Therefore, the application of this material is prohibited in the automotive industry and other applications. It is necessary to develop new materials to replace asbestos as a friction material but still maintain its mechanical properties [12,14,15,16,17].

The different types of friction materials available on the market can be divided into three groups: sintered metals, non-asbestos organic (NAO), and semi-metal (SM). These materials consist of iron powder, steel fibers, graphite, rubber, organic fibers, ceramic, abrasives, lubricants, and fillers. The friction material involves a mixture of several materials bonded together by a phenolic resin (thermosetting) [18]. This review relates to investigating new materials for replacing asbestos and inorganic resins to obtain brake pads with good performance and that are harmless to human health.

Biomass produced from agricultural activities (plant and animal waste products and agricultural residues) is a trending material for the brake pads manufacturing as they are commercially acceptable and environmentally friendly. Palm trees, bamboo, corn stalks, sugarcane bagasse, banana, cashew nutshell, coir (coconut shell), rice straw, pineapple, rice husk, and plants (leaf, stem, fruit, seed, grass, stem, reed) all contain agricultural waste [19]. Agricultural wastes contain a high concentration of natural raw fibers that could be used as reinforcement material in polymer composites. Agricultural waste has a lot of potential in composites because of its high strength, eco-friendliness, relatively inexpensive price, affordability, abundance, and sustainability [20,21]. The chemical and mechanical properties of some agricultural waste materials for composites are shown in Table 1. Due to carcinogenic health issues and the need to enhance the emerging brake pad products’ properties and quality, numerous researchers have employed a wide range of material selections, formulations/compositions, optimization, and processing techniques in multiple research desire to implement asbestos with agricultural-based material in brake pad production [22].

## 2. Factors Affecting the Performance of Brake Pad

### 2.1. Composition of Eco-Friendly Materials

**Figure 3:**
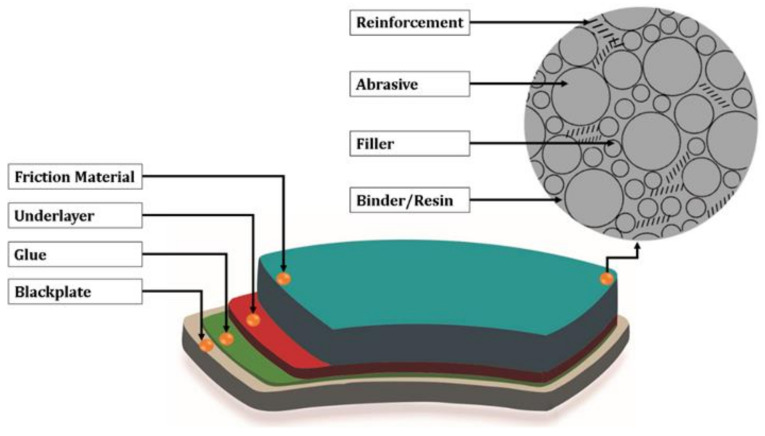
The layers of the brake pad.

Brake pads are made up of several layers (Figure 3). The adhesive that holds the friction material to the other layers is provided by the underlayer, which is placed in between the friction material and the backplate. The underlayer’s primary function is to reduce vibrations caused by friction materials contacting the disc. The backplate gives the brake pads the required rigidity and enables them to keep moving along on the caliper guides. To minimize the amount of unnecessary noise throughout braking, some industries utilize specific interference shims. The friction material that is in direct contact with the disc during the braking process is the essential layer on the brake pads. This material is made of various ingredients that are each developed for specific applications [29,30,31].

The friction material in brake pads consists of binders, reinforcement, fillers, and abrasives (Figure 3). Binders are the materials that keep all of the pads’ components together. This material must have a stable and high friction coefficient, be resistant to high temperatures and fast temperature changes, and be light in weight. Reinforcement is a fibrous material that is added to the binder to increase its mechanical qualities. The brake pads’ durability is greatly influenced by the types of reinforcing materials used. Asbestos is an excellent reinforcing fiber. However, due to its dangerous nature, a replacement material is required. Fillers are utilized to fill the gaps between the brake pads’ other components, while abrasive ingredients are used to modify the coefficient of friction. Steel, refractory oxides, cast iron, quartz, or silicates, for instance, are employed as additives to improve the friction coefficient between the disc and the brake pads due to their hardness. The brake pads’ service life is increased by increasing the friction coefficient [29,30,31].

**Figure 4:**
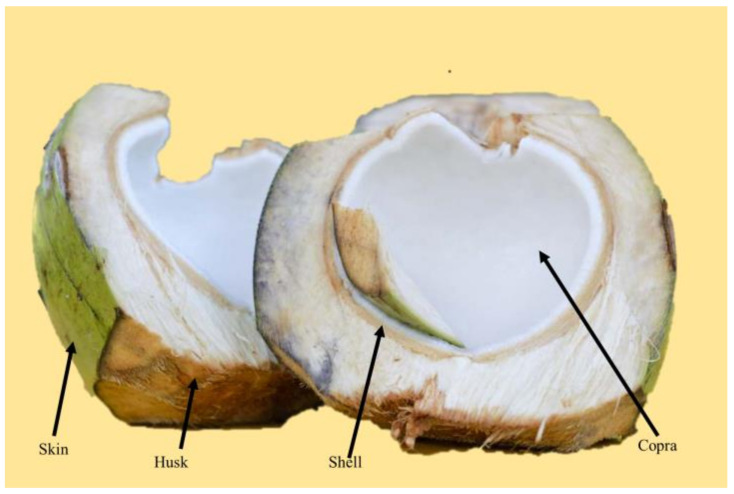
Cross-section of a coconut.

Coconut shell waste is renewable, inexpensive, recyclable, and biodegradable. The coconut shell (Figure 4) has better physical properties and a high compressive strength, which is dependent on the composition of the coconut shell. Based on these characteristics, coconut shell has the potential to be employed as a non-asbestos friction material for non-asbestos brake pads on a lightweight vehicle such as a motorcycle [32,33]. The effect of coconut shell powder on brake friction material was investigated by Daut et al. The specimen is made of a mixture of aluminum oxide, maleic anhydride, epoxy resin, and coconut shell powder with different compositions. In this study, the characterization method used hardness and density tests, each according to ASTM D 2240 and ASTM C 380-79. The optimum formulation was found in the specimens containing aluminum oxide, epoxy resin, coconut shell powder, and maleic anhydride at 49%, 45%, 6%, and 3%, respectively. The hardness, density, and porosity obtained in this specimen were 69.7 Shore D, 1.858 g/cm^3^, and 0.27%, respectively. For friction materials, the resulting mechanical qualities can give a low wear rate and a high friction coefficient. In the manufacturing of brake pads, coconut shell powder can be utilized as an excellent alternative for asbestos [32].

To examine wear analysis of a new advanced eco-friendly non-asbestos friction lining material for automotive drum brake implementations, Shinde et al., employed finite-element analysis, experimental study, as well as microstructural investigations. Prototype drum brake liners made with eco-friendly material compositions were made using an industrial hot compression molding process. Drum brake liners made of phenolic resin (20%), glass fiber and steel wool (15%), coconut shell (30%), powder Al_2_O_3_ + friction dust + Zr_2_SO_4_ (15%), Graphite powder (5%), NBR + Aramid pulp (15%) were used in this study. A tribological performance evaluation on friction lining materials (FLM) was carried out in this study. As a drive system, an electric motor is used. To simulate the wheels of a car, an inertial mass is attached to the brake drum. With the help of load cell adjustment, an electromagnet dynamometer is installed to measure brake torque. The brake force lever is being used to utilize hydraulic pressure to apply the required amount of braking force (33,175 N—−207,294 N). In addition, a finite-element analysis model based on actual operating conditions and various components of the drum brake system has been developed. From these results, it can be seen that the FLM developed using coconut shell powder has good wear resistance. The tribological characteristics of friction lining materials can be studied effectively using the finite-element analysis model [33].

Abutu et al. employed grey relational analysis (GRA) and experimental design via central composite design to manufacture brake pads utilizing epoxy resin (binder), coconut shell (reinforcement), aluminum oxide (abrasive), and graphite (friction modifier). In this investigation, the binder percentages, reinforcement material, friction modifier, and abrasive were 35%, 52%, 5%, and 8%, respectively. When compared to commercially available samples, the optimized coconut shell-reinforced brake pad had a beneficial effect. It was able to produce less brake noise and vibration during usage, according to the findings of this investigation. In addition, curing time has the greatest impact on the coconut shell-reinforced friction material’s ultimate tensile strength and hardness, accounting for 31.40% and 30.38% accordingly. In contrast, heat treatment time has the greatest impact on the coconut shell-reinforced brake pad’s friction coefficient and wear rate, accounting for 24.23% and 46%, accordingly [34].

To produce brake pads, Bashar et al., used coconut shells as filler instead of asbestos. In this research, iron chip, epoxy resin, cobalt nephthanate, methyl ethyl ketone peroxide, brass, and silica, were employed as reinforcement, binders, accelerator iron, catalysts, friction modifiers, and abrasives, accordingly. The wear test was conducted on specimens gauging 14 × 7 × 8 mm and of various compositions and models. Each sample (which is rigidly clamped) is placed together with the grinding disc for 5 s. Before and after grinding, the samples were weighed. The weight difference between each sample indicates a weight loss. The disc diameter and speed of the grinding machine are 6600 rpm and 210 mm, respectively. The higher the amount of ground coconut powder, the lower the compressive strength, breaking strength, impact, and hardness, showing that a large percentage of ground coconut powder induces brittleness. The qualities of cold-worked composites are identical to those of Honda commercial brake pads from the Enuco model, demonstrating that the materials chosen are appropriate. With 10%, 50%, 0.50%, 20%, 10%, 0.50%, and 9% of reinforcements, matrix, catalyst, filler, abrasives, accelerator, friction, and modifiers, specimen 4 had the lowest wear rate [35].

**Figure 5:**
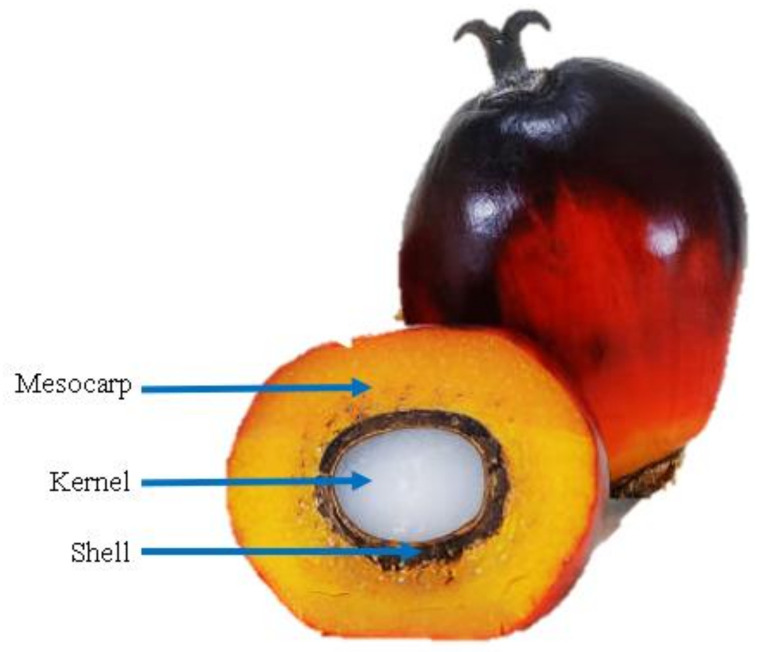
Cross-section of a palm.

High-quality non-asbestos brake pads from palm kernel shell (PKS) (Figure 5) and coconut shell (CNS) have been investigated by Egeonu et al. For the disc brake friction lining with Mitsubishi L-300 geometrical standards, PKS and CNS powders were utilized as the foundation material, metal chips, and carbides as the abrasives, graphite as the lubricant, and polyester resin as the binder. Three separate samples were developed by altering the mass compositions of CNS and PKS. The mass of PKS (36 g) and CNS (36 g) in Sample A is equal. Sample B has a higher PKS mass (54 g) and a lower CNS mass (18 g), whereas Sample C has a lower PKS mass (21 g) and a higher CNS mass (51 g). On the other hand, the composition of the binder, lubricant, and abrasive were all kept constant. A friction testing machine was created using a lathe, a wheel disc, and a fabricated brake pad holder. The disc was tightly gripped in the lathe’s chuck, which was constantly moving at 180 rpm. Based on spring parameters and lining contact area, the linings/disc pressure was set at approximately 56.08 kN/m^2^. For 15 min, the disc was spun at the specified speed. The wear was determined by comparing the mass of the brake pads before and after the test run. Sample C had better mechanical properties than the other specimens. The findings of this research, nevertheless, showed that commercial brake pads wear out faster. Since various sorts of binders are used, this occurs. Commercial brake pads use phenolic resin, which will decompose at about 450 °C. In this study, the authors used polyester resin, which will decompose at a temperature of 250–300 °C [36].

Juan et al., employed a hydraulic press with a pressure of 15 KN/m^2^ for 4 h to create brake pads using various compositions of coconut shell powders, candlenut shell powders, pineapple fiber leaf, iron sand, carbon, and polyurethane resin. The highest hardness was found in the S-03 sample, which had a composition of CNS (15 wt.%) and CdNS (25 wt.%) and 92 HRK value. The highest hardness value resulted in this specimen producing a lower wear rate than other specimens. Under dry sliding conditions at room temperature, the composites’ wear performance was assessed using the pin-on-disc method. The specimen remains stationary for 120 s while the disc rotates and normal force is applied through the lever mechanism. The wear rate of this specimen is 3.67 × 10^−5^ g/mm^2^s. Meanwhile, the sample S-03 has 5.84 × 10^−3^% water absorption after the water absorption test. Overall, the tests showed that the material used in brake pads provides less dusting, a brake pad that lasts longer, safety, and enhanced stopping performance. In addition, the test findings demonstrate that samples with a higher hardness possess greater wear resistance. Moreover, less resin and higher density lead to quieter, more consistent braking performance as well as clean braking [37].

Moreover, Kholil A. et al. investigated brake pads made from sawdust, coconut fiber, and cow bones. The polyester resin was used as a matrix in this study, with a constant composition of 50%. To obtain a fine material, the coconut fiber, sawdust, and cow bones were ground and filtered through a 40-m mesh sieve before use. Wear testing is carried out to obtain wear data after testing braking at speeds of 10, 15, and 20 km/h. The brake pads before and after the test were weighed to determine the mass loss. Furthermore, the coefficient of friction of each test object is determined with the help of an inclined plane (90°). Specimen C, which was made up of 50% polyester resin, 20% wood powder, 20% coconut fiber, and 10% cow bone, had hardness characteristics and braking time that was similar to commercial products. Furthermore, specimen C has a rougher surface than the other specimens, improving braking performance and wear resistance. According to this study, the right composition of coconut fiber, sawdust, and cow bone could be used for motorcycle brake pad materials [38]. CaCO_3_ is the major component of cow/bovine bones, and it is widely used in the production of bioceramic and biocomposite materials [39,40,41,42].

As demonstrated by Rajmohan et al., an eco-friendly natural fiber-based composite comprised of sugar cane, CNS, epoxy resin, and SiC powder could be employed as a substitute medium to asbestos in the brake pads fabrication. In this study, tribological performance was carried out by the pin-on-disc method. Since the friction coefficient increases as the content of coconut shell powder increases, the wear rate decreases. Furthermore, the friction coefficient of sample 1 with a composition of CNS (6 g), sugar cane (2 g), SiC powder (0.5 g), and epoxy resin (20 g) was higher than the asbestos-based brake pads [43]. Because of the cellulose content of 34% in coconut shell powder, it has better water absorption and wears resistance. Cellulose is a natural product that comes from trees. This material is most often used in powder form for the production of brake pads. Cellulose in brake pads is usually burned at high temperatures during production. This produces a porous material, lowering the finished product’s density and reducing braking noise. A friction material containing 10 wt.% microcellulose fiber shows comparable results to commercial brake pads because its wear rate and coefficient of friction are 3.7 mg/m and 0.357, respectively. In addition to this, the micro cellulose fiber content of 10 wt% leads to the excellent thermal stability of the prepared composite samples up to 500 °C [30,43].

Sutikno et al. used bamboo fiber and coconut to create a non-asbestos brake pad material. Alumina, epoxy resin, and magnesium oxide are among the other components employed in this study. Brake pads are made at 200 °C for 20 min under a 1000 kgf compression load. The Rockwell hardness test is carried out according to ASTM E18-15. Wear tests are carried out using a Pin-on-Disc Tribometer in accordance with ASTM G99-95A to determine the specific level of wear on the composite brake pads. Non-asbestos brake pads made of bamboo fiber, epoxy, magnesium oxide, and aluminum oxide with a composition of 29%, 40%, 6%, and 25%, respectively, had a hardness of 37.14 HRB, temperature resistance of 251.53 ℃, a friction coefficient of 0.454, and a wear rate of 0.323 mm^3^/N·mm, accordingly. While the brake pads are made of 20% coconut fiber, 46% epoxy, 6% magnesium oxide, and 28% aluminum oxide, they have a hardness of 44.10 HRB, temperature resistance of 250.56 ℃, a friction coefficient of 0.46, and a wear rate of 0.242 mm^3^/N·mm. The wear rate and friction coefficient of bamboo fiber-reinforced composite brake pads are comparable to commercial brake pads. Although commercial brake pads have a greater wear rate and friction coefficient, coconut fiber-reinforced composite brake pads have a higher wear rate and coefficient of friction. The non-asbestos brake pad, on the other side, has a lower hardness than commercial brake pads in this study [44].

Pujari et al. studied the influence of the volume fractions of Nile rose, palm kernel, and wheat powder on the wear parameters of non-asbestos brake pads. The Nile rose (0–15%), palm kernel (0–50%), and wheat (0–10%) is used to make brake pads, together with aluminum oxide (5–20%), graphite powder (10–35%), and phenolic resin (35%). The phenolic resin was chosen because it produces good bonding properties with the fiber. In this study, the specimens were prepared using the hand lay-up method. Composite wear characteristics are measured using a wear testing machine according to the ASTM G-99 standard. The research’s findings suggest that the PKS could be used in place of asbestos as a friction material. The specimens with the best wear and hardness properties were those composed of 50% palm kernel (specimen 5 on Type-1 composites). Al_2_O_3_, palm kernel, phenolic resin, and graphite are used in this specimen, and their compositions are 5%, 50%, 35%, and 10%, respectively. PKS particles improve abrasion resistance, thermal stability, and sliding wears resistance while also delaying the transition from mild to severe wear [45].

The addition effect of kenaf fibers on the quality of eco-friendly brake pads was studied by Madeswaran et al. Kenaf fiber is one of the natural fibers that will increase the heat resistance and strength of the brake pads because it has high strength and good heat resistance. Aerosil and aramid fibers are used in this study to reduce pores in the brake pad. The stopping performance, friction coefficient, stopping performance, and wear resistance are all improved with alumina and zirconium. Graphite was used in this study as a solid lubricant with good thermal resistance properties. Furthermore, phenolic resin is used in this study because it has a good ability to bind other materials and fibers. A hydraulic hot press machine was used to compact the ingredients for 30 min at a pressure of 20 MPa and a temperature of 120 ℃. The wear characteristics of this research are measured using the pin-on-disc method according to the ASTM G-99 standard using wear testing apparatus (TR-20LE-M108). This research showed that brake pads made of Kenaf fiber, Phenolic resin, Graphite, Alumina, Zirconium, Aerosil, and Aramid had compositions of 25%, 40%, 8%, 3%, 2%, 10%, and 12, respectively. Note that 10% can be used as efficient brake pads. A specimen with this composition produces a constant friction coefficient even at different speeds. It reduces wear so that eco-friendly brake pads that use this composition can be employed to substitute asbestos brake pads in the future [46].

Coconut fiber, banana fiber, and rice husk powder have been used by Subramani et al., to manufacture bio-composite brake pads with varying fiber percentages. Brake pads are made using a basic wet hand lay-up procedure with the ingredients of banana fiber (5 and 10 wt.%), coconut fiber (2 and 5 wt.%), rice husk (3 and 5 wt.%), aluminum oxide (15 wt.%), graphite powder (30 and 35 wt.%), as well as epoxy resin (35 and 40 wt.%). The pin-on-disc method is used to evaluate the wear characteristics of the composite using a wear testing machine according to the ASTM G-99 standard. Sample A has a lower friction coefficient than composite Sample B. Furthermore, composite Sample A has a higher hardness than composite Sample B. Sample B has a lower percentage of natural fibers and a higher percentage of graphite and resin. Young modulus and compressive strength will both improve with more fiber content. Because hardness is a fiber volume’s function, like fiber content increases, so does hardness. With increasing fiber content, the wear rates of Sample A composites rise. When the speed is set to 350 rpm, though, Sample A and Sample B show lower wear rates than commercial brake pads [47].

Reviewing the literature, the filler type effect with varied concentrations utilized for the non-asbestos brake pads production is indicated in Table 2. The asbestos brake pads’ friction coefficient is 0.3–0.4, and the wear rate is 3.8 mg/m [45,46]. Using agricultural waste or natural fiber to make non-asbestos brake pads has a major influence on wear rate and friction coefficient. This occurs because the use of various concentrations of agricultural waste can produce a wear rate as well as friction coefficient that is similar to asbestos brake pads [48]. Table 2 suggests that different types of agricultural waste with the right composition can be used as brake pad fibers. In addition, a better wear rate is also influenced by the proportion of materials and the right bonding properties. Natural fibers’ excellent adherence to resin gives good wear resistance for composites [45,48,49,50,51].

### 2.2. The Particle Size of Eco-Friendly Materials

The manufacture of brake linings for Toyota Camry 2000 using palm kernel and coconut shell powder was studied by Anaidhuno et al. (2017). As foundation materials, the authors employed epoxy resin as a binder, palm kernel, and coconut shell powder, and carbon as a fiber reinforcement. Abrasives included copper, zinc, aluminum, and cashew nut shells, as well as shoe rubber dust as filler. The coconut and palm kernel shells were pulverized into grit/mesh sizes of 0.25 mm, 0.35 mm, 0.45 mm, 0.55 mm as well as 0.65 mm, accordingly. The pad material had a friction coefficient of 0.4–0.65, a bonding strength of 25–27 kg/cm^2^, a scratch hardness of 80–85, and a wear rate of 0.02–0.06 mm/min, based on the experiment’s outcomes. The commercial brake pad material hardness is 80–85, the bonding strength is 25–27 kg/cm^2^, and the wear rate is 0.03–0.08 mm/min. The large particle size (grit size) creates an open structure bond, which allows the particles to easily break away from the bond when force or pressure is applied to the brake pads. Small grit (small particle size) creates a stronger structural bond and allows the particles to withstand more force or pressure before breaking. As a result, samples with particle/grit sizes of 0.25 mm and 0.35 mm provide better/acceptable wear resistance and improve bond strength properties [52].

The development of non-asbestos brake pads using sawdust was investigated by Oladokun et al. Sawdust from hardwood is sieved into 100 μm and 250 μm grades for the brake pads production. Brake pad samples contained 40, 45, 50, 55, and 60% sawdust. Resin as a binder, silicon as an abrasive, steel dust as a lubricant, and carbon black (reinforce) were among the other components employed in this study. The samples were then dried at 160 ℃ in a hot press at five different printing pressure loads: 10 Mg, 20 Mg, 40 Mg, 60 Mg, as well as 80 Mg. In all specimens, there was a decrease in wear rate with a decrease in sawdust particle size. This occurs due to the smaller particle size, which leads to greater density and strong bonding with the other constituents. Moreover, smaller particle size increases surface area, which improves the sawdust particles’ capacity to attach to resin. The smaller sawdust particle size gave the highest density, hardness, and compressive strength values and improved the brake pad specimen’s wear properties. In addition, the wear rate decreases as the mold pressure increase from 10 to 80 Mg. The wear rate on the specimen with the pressure of the mold of 80 Mg is proportional to the level of wear on the asbestos brake pads [53].

Elakhame et al. (2014), who produced new brake pads employing varied particle sizes of PKS, backed with the findings of this study. In this study, PKS, resin, graphite, steel, and SiC were used to produce brake pads with compositions of 35–55%, 20%, 10%, 15%, and 0–20%, respectively. The manufacture of brake pads is carried out through compression molding. According to the conclusions of this investigation, for all material compositions, the sample with 100 μm of palm shell had better properties than other samples with particle sizes of 1 mm, 710 μm, and 355 μm. Furthermore, as the sieve size was increased, the produced sample’s compressive strength, hardness, density, and porosity decreased. Meanwhile, the larger the particle size, the greater the absorption of water and oil, the burnt proportion, and the wear rate. The research’s findings suggest that palm shells could be a viable alternative to asbestos in brake pad manufacturing [54].

The impact of rice husk particle size on the performance of resin-based brake pads was examined by Nandiyanto et al. Rice husk with different particle sizes (250, 500, and 1000 μm) was mixed to a resin produced from cycloaliphatic amine and Bisphenol A-epichlorohydrin combination in this experiment. The brake pad was made by combining 22 g of resin mixture with 13 g of rice husks. After that, the liquid was put into a silicone mold and left to dry at room temperature for two days. The particle size of the rice husk particle–resin matrix affects interaction distances, interfacial bonding, and thermal softening. By lowering the particle size, the brake pad’s compressive strength can be improved. Reduced particle size and also less mass loss meant fewer pores, a higher friction coefficient, better wear rate, and a coarser brake pad surface. Agro-waste, such as rice husks, can be utilized as a substitute for friction material in brake pads [55].

Research conducted by S.G. Amaren et al. shows the wear rate on non-asbestos brake pads is influenced by the particle size of periwinkle shells as fillers. In this study, periwinkle shells were dried, ground, and sieved using a sieve size of +710, +500, +355, +250, and +125 μm. The asbestos-free brake pad was made by changing the size of periwinkle shell particles (ranging from +125 to +710 μm) and binding them with phenolic glue. After homogeneously combining 35 wt.% phenolic resin and 65 wt.% periwinkle shell particles for a while, the mixture was transferred to a mold at 160 ℃ for 1.5 h under 15.5 bar pressure. A post-cure at 140 ℃ for 4 h was performed on each specimen. The wear rate increases with increasing sliding speed, temperature, load, and periwinkle particle size, according to the research [56,57]. The friction coefficient achieved is within the reference values for brake pads for automobiles. From this research, the best wear resistance was found in the smallest periwinkle particles (+125 μm). As the pore size grows, the interfacial area grows, causing poor interfacial bonding and ineffective stress transfer between the resin and the particles. Smaller particles have higher total surface energy for a given particle loading. Wear is minimized as particle surface area grows due to a more effective stress transmission mechanism.

The lower wear rate of the periwinkle-based brake pad composites could be explained by the harder materials’ stronger load-bearing capacity and a strong interfacial connection between the particle and the resin, which minimizes the probability of particle pullout and hence higher wear. The wear rate of these brake pad composites is well within the automotive standard ranges for brake pad manufacture, with periwinkle shell particle sizes ranging from +125 to +250 μm. Periwinkle shell particles can effectively replace asbestos in brake pad manufacturing, according to the findings of this study [56]. The results of this study are supported by research conducted by Dagwa et al., who developed non-asbestos brake pads from PKS [58]. The decrease in palm shell particle size caused the increase in friction coefficient, according to the findings [58,59]. Reduced wear rate on the brake pads due to the reduced particle size of the filler [59,60,61,62,63,64].

In addition to affecting the mechanical properties and wear resistance, particle size also greatly affects the brake resistance to fade. Tamo et al. studied the influence of PKS particle size on the wear and brake pads’ fade resistance. In this study, the PKS used had particle sizes of 0.212 mm, 0.300 mm, 0.42 5 mm, 0.600 mm, and 0.850 mm. PKS (12.5% wt.) is mixed with other materials and processed using cold pressing, hot pressing, and post-curing methods to produce brake linings. Compared to other specimens, the best density, fading resistance, and wear were achieved utilizing PKS with a particle size of 0.300 mm [65].

### 2.3. Types of the Binder for Brake Pad

The binder resin is assumed to serve as a key influence in brake performance since it keeps components together as a matrix in a composite [66,67]. The kind of binder and its proportion in the brake pad material composition are crucial to meeting the standard requirements for friction materials [67,68]. Phenolic resins are thought to be the first production of commercial polymeric products made from simple low-molecular-weight compounds. The physical and mechanical properties of the phenolic resin are shown in Table 3. Phenolic resins are suited for high-temperature applications that require parts to be fire-safe. Phenolic resins are used in various applications, including ballistics, electronics, offshore water pipe systems, mine ventilation, aircraft, mass transit, and rail. Low thermal conductivity, excellent corrosion, low density (weight-efficient), chemical resistance, enhanced design flexibility, outstanding fatigue and impact properties, cost-effective production of complex three dimensional (3D) structures, radar/sonar transparency, enhanced acoustic performance, and low maintenance are just some of their primary elements [69,70,71,72]. The phenolic resin has already been utilized in brake pads due to its superior heat resistance and inexpensive manufacturing cost. Nevertheless, recent high expectations for brake performance have compelled enhanced friction stability and wear resistance at higher temperatures, necessitating various chemical modifications to the binder resin to fulfill the needs for a short stopping distance and lowered pad wear in brake applications at higher temperatures [67,68]. High thermal stability brake pad friction materials result in safer braking performance and better resistance to thermal decay. The friction material of the brake pads has increased thermal stability, resulting in an improved ability to withstand compressive forces during the braking process and a stable friction coefficient. As a result, the amount of weight loss and the rate of wear on the brake pads are reduced. In general, brake pads with a high coefficient of friction provide better braking with less effort on the brake pedal [73,74].

Phenolic resin contributes to the friction performance of the brake pads. The findings of the Jang et al.’s study revealed that a considerable amount of phenolic resin led to an unstable friction coefficient due to phenolic resins’ poor temperature stability [80]. The amount of phenolic resin with cashew nut shell liquid (CNSL) and potassium titanate enhances the hardness and friction coefficient at temperatures around 100 ℃ [81]. The friction coefficient was reduced as the amount of CNSL-modified resin was increased, however, the mechanical characteristics and the wear rate were enhanced. The optimum percentage of CNSL resin for best friction performance in extreme operating conditions was 10 wt.%. The CNSL phenolic resin modification performance was degraded in terms of fade and recovery [82].

P. Nawangsari et al. investigated the effect of phenolic resin as a binder in a non-asbestos organic brake pad with varying volume fractions of 25 wt.%, 20 wt.%, 15 wt.%, and 10 wt.%. Phenolic resin, BaSO_4_, friction dust, Al_2_O_3_, SiO2, MgO, copper, MoS_2_, graphite, and hBN were used in this study. The brake pad composite was made using powder metallurgy. The compaction pressure was 47 MPa, the molding temperature was 150 ℃, and the post-curing temperature was 130 ℃, with a holding time of 6 h. As the volume percentage of phenolic resin grew, porosity reduced, but hardness increased. This is because, during the hot press process, phenolic resin particles fill the empty region, increasing the contact area between particles. As a consequence, the bonding strength and hardness of the composite sample increase, whereas the porosity of the composite sample reduces. The composite samples’ thermal stability reduces as the phenolic resin volume fraction grows. Thermal stability is better in the composite sample with 10% phenolic resin than in the other specimens. With an increase in the volume percentage of phenolic resin, the friction coefficient will decrease, and the volume wear rate will rise. Moreover, while having superior thermal stability than the other specimens, composite samples comprising 10% and 15% phenolic resins were not recommended for use in the production of this brake pad since they have a poorer resistance to mechanical stress throughout friction performance tests [83].

Binda et al., described a unique friction element made of slate particles used as a tribological reinforcement in a phenolic resin-based composite matrix. In this study, fiberglass, aluminum oxide, and graphite were used at a constant mass percentage. At the same time, slate particulates and phenolic resins are used in varied compositions. The materials were placed in a cylindrical metal mold and pressed for 12 min at 160 ℃, with pressures hitting 20 MPa; pressure relief intervals were used to allow the composite to degas from the crosslinking reaction of the resin. Lastly, to attain a higher degree of cure, the molded pellets were kept at 200 ℃ for 3 h. The composites’ friction coefficients were found to be regular and stable, averaging 0.44 between samples. When compared to commercial friction materials currently in use, the optimized formulation of 40% slate and 35% phenolic resin (specimen A3) had the most desirable characteristics. This specimen employs slate with the lowest composition and the highest resin composition. This formulation resulted in a composite that performed admirably in friction testing and even better in wear tests. As a result, it was the greatest alternative for a low cost-to-benefit ratio. This is especially relevant in light of the brake rotor’s low wear rate, which is, in general, the most expensive component of a friction-based system [84].

Yanar et al., investigated the phenolic content influence in brake pad composites on the fade, friction, and wear parameters. The brake pads utilized in this experiment are made up of six different materials. Only the binder resin and barite filler (BaSO_4_) concentrations changed, but the concentrations of other elements including Kevlar, rock wool, magnetite additions, and graphite remained unchanged (total 50 wt.%). The bonding resin proportions in the composite matrix are 16 wt.%, 17 wt.%, 18 wt.%, and 20 wt.%, accordingly. The barite percentages are 34, 33, 32, and 30 wt.%, respectively. The components were combined together and squeezed at 50 bars inside a pre-heated 90 ℃ compression mold. The mold’s temperature was then increased to 150 ℃, and the samples were cured in the mold for 10 min at a pressure of 50 bar. To confirm that all of the resin in the mixture had cured, the specimens were post-sintered for 20 min at 200 ℃. Reduced resin concentrations lead to better friction, wear resistance, and lower mechanical characteristics. Composite samples with higher resin content showed the lowest wear resistance owing to the development of secondary plateaus on the wear surfaces of the investigated samples [85]. Subagia et al. also reached the same conclusion [86]. According to the author, the higher the phenolic resin content, the higher the wear rate on the brake pads due to the matrix becoming less bound to the powder [86,87].

The influence of phenolic resin content on brake pad performance with banana peel waste was investigated by Idris et al. The banana peels were dried and processed into banana powder in a ball mill at a speed of 250 rpm (uncarbonized, BUNCP). The powder was put in a graphite crucible and burned at 120 ℃ in an electrically powered furnace to generate banana peel ash (carbonized, BCp). Resin composition ranged from 5% to 30% by weight. Before being transferred to a mold in a hot platen press for 2 min at 150 ℃ and 9.81 × 10^7^ N/m^2^ pressure, the composition was thoroughly mixed in a mixer to obtain a homogeneous mixture. The brake pad composition was baked for 8 h at 130 ℃ in an oven. The results of this experiment demonstrated that appropriate bonding could be achieved with uncarbonized banana peel particles (BUNCp) at 20 wt.% resin additions, but not with carbonized banana peel particles (BCp) at 30 wt.% resin additions. The compressive strength, hardness, and specific gravity of the generated samples all increased as the weight percentage resin was increased. However, the oil soak, water soak, wear rate, and percentage burned decreased. The wear rate also decreased when the weight percentage of resin in the banana peel particles increased. This could be because the microstructure is more dense, resulting in stronger banana peel-resin bonding. In addition, the hardness and compressive strength of the samples rose as the amount of resin added to the banana peel particles increased. The brake pad composition’s decreased wear rate can be attributed to the formulation’s higher load-bearing capacity and strong interfacial bond between the particle and the resin, which decreases the risk of particle pullout, leading to increased wear. The BCp formulation’s elevated wear rate may be explained by the low interfacial bonding strength values achieved [88].

In addition to the amount of resin used, the type of resin used can have an impact on the brake pads’ performance. The amount and type of binder are both critical for modifying the required performance properties of the friction material on the brake pads. The wear resistance, friction coefficient, and thermal resistance of the friction specimen are all influenced by the binder matrix. Furthermore, during brake application, the polymer matrix limits noise propensity. Interestingly, the binder resin has a big influence on the friction material fabrication design parameters [89,90,91].

Joo et al. looked at the tribological behavior of brake linings and found that different binder resins had distinct effects. The brake lining specimens in this investigation were made using a variety of binder resins. This experiment used alkyl-modified phenolic resin, straight phenolic resin, acrylic 30% modified phenolic resin, silicon-modified phenolic resin, and aromatic ring-modified phenolic resin. Table 4 shows the thermal characteristics of the resins employed. The brake linings were created using a combination of mixing, performing, compression molding, and heat treatment. The tribotester findings demonstrate that at high temperatures, the decomposition of the binder resin has a substantial impact on the friction coefficient, wear rate, and brake linings’ brake emission. The use of heat-resistant resins in brake linings resulted in a considerable reduction in particulate matter with a diameter of less than 2.5 m (PM2.5) at high temperatures, demonstrating that heat-resistant binder resins can reduce brake lining wear rate and particle emission. Despite having a low high-temperature wear rate, the heat-resistant alkyl-modified resin brake lining generated a substantial amount of brake emission, showing that the unanticipated volatile vapors released by the modified resin can boost brake emission. A high-temperature wear test’s activation energy is much higher than a low-temperature wear test. The binding resin’s activation energy used to create brake lining specimens at high temperatures varies greatly, implying that the heat resistance resin has a major effect on the brake lining’s wear rate [66].

The higher the thermal breakdown/degradation temperature in resin-based brake pad materials, the lower the oxidation and decomposition. The brake pads’ friction qualities will be stable as a result. Various modified phenolic resins (modified with cashew nut shell oil, melamine, nitrile rubber, and so on) can be used to raise the resin’s decomposition temperature. In addition, the fade and wear of the friction materials were linked to the binder resin’s heat degradation. The percentage of fade was significantly reduced in friction materials with changed resin. High-thermal-stability friction materials showed resistance to fading [91,93].

Ozturk et al., investigated the fiber length effect and resin type on the mechanical properties and friction characteristics of brake friction materials. Straight phenolic resin (SR), melamine resin (MR), and cashew nut shell liquid modified resin (CR) were used as matrix materials in their study. As an inorganic fiber, several lengths of Lapinus were employed. Both fiber length and resin type influenced the friction materials’ mechanical and tribological properties, according to the findings. The SR and MR composites had the highest and lowest coefficients of friction, accordingly. MR and CR composites have the highest and lowest wear resistance, correspondingly. On the other side, increasing the fiber length raised the wear resistance of the composite [92].

Additionally, Kim et al., investigated the friction and wear properties of two phenolic resins commonly utilized in automobile friction materials. They employed a straight novolac resin and a modified novolac resin as binder ingredients in this research. Furthermore, six distinct friction materials, each with a different combination of phenolic resins and aramid pulp, were produced and evaluated. Two independent test modes were employed to study friction characteristics in terms of friction heat and cumulative thermal history. The type of phenolic resin utilized and the ratio of resin to aramid pulp had a big impact on friction qualities like friction stability and wear resistance. Friction materials developed from modified novolac resin have more stable friction qualities than those made from unmodified novolac resin. Regardless of resin type, friction materials reinforced with 10% aramid pulp demonstrated a considerable improvement in friction stability. On the other side, the friction materials with the changed resin exhibited a considerable reduction in wear resistance [94].

## 3. Discussion

Various agricultural wastes were researched and employed as asbestos substitutes in brake pads in this research. Thus, it was found that the brake pads developed with agricultural waste perform almost the same as the brake pads developed with asbestos. Furthermore, the use of agricultural waste on brake pads can reduce environmental pollution and health risks. Brake pads are crucial components of the braking system. Brake pads are typically made of friction material that is supported by steel plates. Friction materials must maintain a higher and optimal coefficient of friction during the braking process than other tribo-materials. Friction material wear is primarily caused by adhesion and abrasion and typically occurs due to the formation and peeling of the friction layer, which holds the contact load and thus affects friction behavior. There are two plateaus in the friction layer: primary and secondary plateaus. The hard part of the brake pads makes contact with the disc during brake pad-to-disc contact, creating micro-grooves in the disc and allowing wear fragments to flow continuously. The combination of temperature, pressure, and tangential forces causes the fragments to be compressed and compacted against the existing plateau, ensuring that the compaction of the fragments is heterogeneous. In contrast, the smaller fragments escape and are released into the atmosphere as worn flakes or wear debris [95,96,97,98].

The initial wear causes particles to be released from the matrix (resin), resulting in the formation of a prevalent primary plateau. The formation of the plateau is a dynamically developing process rather than a stationary one. Steel fibers and high-tensile hardened copper or brass particles are usually involved in the formation of these plateaus, which are typically larger than 100 µm in length and have hardness values that exceed those of the disc material. Further to that, depending on the contact stresses and shear stresses involved, micrometric and submicrometric wear particles tend to accumulate near the plateau, where they are also compacted to some large extent. As a result, a secondary plateau is formed, consisting of small, compacted particles that result from pad and disc wear. In this regard, the “primary contact plateau,” namely, the part of the material on the pad’s surface that is more resistant to wear, is distinguished from the “secondary contact plateau,” which is more resistant to wear [97,98,99].

The plateau is damaged and forms debris, primarily from the secondary plateau, due to strong pressure and tangential forces. Loss of support from loose fibers due to wear, irregularity or dirt on the disc, abrasion, and adhesion of the three bodies are all causes of damage. The main cause of wear particle fragments being released into the atmosphere is the breakdown of the friction layer. Wear occurs in brake pad systems during both normal and heavy braking. Furthermore, debris is formed due to the three bodies’ abrasive wear caused by the degradation of the filler particles from the organic binder, which is aided by local heat generation in the asperities (morphology) [97,98,99,100].

The friction force, speed, duration of contact, and shear strength all influence brake pad wear to make braking temperature, material type, geometry, load history, and friction material topography [95,96,100]. In general, the effect of braking pressure on friction material wear and wear behavior can affect the actual contact area of the brake pads and disc, the formation of the friction film, friction material components, and structures, as well as changes in the type of wear that occurs. The wear of the brake pads surface will be affected by the rise in temperature. The asperity temperature may be much higher than the surface temperature, resulting in a high-temperature local zone. The formation of the primary plateau is correlated with an increase in friction as time and temperature increase. The fibers are damaged, and the particles on the surface become harder. The primary plateau is represented by the composite, while the secondary plateau is represented by the smooth patches that protrude from the surface. The heat generated by friction during braking can easily raise the frictional interface temperature above the binder resin’s decomposition temperature, causing a change in frictional force during braking. With changes in the sliding speed and the brake pads material ingredients, the friction material’s wear rate changes frequently. Note that with an increasing sliding speed and temperature, the specific wear rate of the brake friction material was found to increase. Because of the high roughness (asperities) in the brake pad surfaces, the rate of repeated impact loading increases while the sliding speed increases. This loading increases frictional thrust, causing local vibrations and crackling at the sliding surface’s interface, resulting in delamination and fiber fracture [97,98,99,100].

In this review article, the focus is on the eco-friendly brake pad’s performance affecting factors. A better wear rate is also influenced by the proportion of materials and the right bonding properties. The strong adhesion of natural fibers with resin provides good wear resistance for composites [45,49,50,51]. In addition, reducing the level of wear on the brake pads can be done by reducing the particle size of the filler because smaller particle size will increase the bond between the binder and the filler [59,60,61,62,63,64]. Furthermore, the amount and the type of resin used can have an impact on the brake pad’s performance. The amount and type of binder are both critical for modifying the required performance properties of the friction material on the brake pads. The wear resistance, friction coefficient, and thermal resistance of the friction specimen are all influenced by the binder matrix [89,90,91]. The brake pad’s wear rate and particle emission can be reduced using heat-resistant binder resins. The greater the heat resistance of the resin, the higher the thermal decomposition temperature, thereby reducing decomposition and oxidation. As a result, the friction properties of the brake pads will be stable [91,93].

## 4. Conclusions

- Various agricultural wastes were researched and employed as asbestos substitutes in brake pads in this research. Thus, it was found that the brake pads developed with agricultural waste perform almost the same as the brake pads developed with asbestos. Furthermore, the use of agricultural waste on brake pads can reduce environmental pollution and health risks. The use of agricultural waste or natural fiber to make asbestos-free brake pads affects the wear rate and friction coefficient significantly. This is because varying the amount and composition of agricultural waste can result in a similar wear rate and friction coefficient as asbestos brake pads.
- Brake pads made from agricultural waste need to pay attention to the type, amount, and particle size of the agricultural waste used to have better physical and mechanical properties. In addition, the type and amount of resin also have a decisive effect on the quality of the brake pads. The amount and type of reinforcement are important in changing the performance characteristics required for brake pad friction materials. According to the results of this review, an increase in the percentage or amount of agricultural waste results in a decrease in the breaking, compressive, hardness, and impact strength of the brake pads.
- The large particle size of filler or reinforcement creates an open bond structure that allows the particles to easily disengage from the bond when force or pressure is applied to the brake pads. The small particle size of the reinforcement creates stronger structural bonds and allows the particles to withstand more force or pressure before breaking. The hardness, compressive strength, porosity, and density of the resulting sample decreased with the increasing sieve size used. Meanwhile, brake pads produced with large particle sizes will result in a higher percentage of oil and water absorption, wear, and burnt parts.
- The use of phenolic resins for brake pads based on agricultural waste has improved physical, mechanical, and tribological properties over polyester resins. This is because the decomposition temperature of phenolic resin is higher than that of polyester resin. A high decomposition temperature will result in better wear and fade resistance, thereby increasing the lifetime of the brake pads. In addition, brake pads with modified phenolic resin will give better results. The heat resistance of the resin has been improved by the modification process by adding additives to the phenol resin. This raises the temperature of the decomposition and will produce a higher and more stable coefficient of friction.

## Figures and Tables

**Table 1:** Chemical and mechanical properties of some agricultural waste materials for composites [19,23,24,25,26,27,28].

	Chemical Properties				Physical and Mechanical Properties			
Agricultural Waste Type	Cellulose (%)	Hemicellulose (%)	Lignin (%)	Wax (%)	Density (g/cm^3^)	Elongation (%)	Tensile Strength (MPa)	Young’s Modulus (GPa)
Jute	45–71	13.6–21	12–26	0.5–2	1.3–1.46	1.5–1.8	393–800	10–30
Ramie	68.6–76.2	13.1–16.7	0.6–0.7	-	1.5	2.0–3.8	220–938	44–128
Sisal	67–78	10–14.2	8.0–11	-	1.33–1.5	2.0–14	400–700	9.0–38.0
Kenaf	31–39	21–21.5	15.9	-	1.2	2.7–6.9	295	-
Abaca	60.8–64	17.5–21	12–15.1	-	1.5	-	980	-
Hemp	57–77	14–22.4	3.7–13	0.8	1.48	1.6	550–900	70
Flax	71	18.6–20.6	2.2	-	1.4–1.5	1.2–3.2	345–1500	27.6–80
Coconut (coir)	36.62–43.21	0.15–0.25	41.23–45.33	-	0.67–1.15	27.21–32.32	173.5–175.0	4–6
Bamboo	73	12	10	-	0.6–1.1	4–7	360.5–590.3	22.2–54.2
Sugarcane (Bagasse)	55.60–57.40	23.90–24.50	24.35–26.30	-	0.31–1.25	6.20–8.2	257.3–290.5	15–18
Pineapple	70.55–82.31	18.73–21.90	5.35–12.33	-	1.25–1.60	2.78–3.34	166–175	5.51–6.76
Palm kernel shell (PKS)	31.33	17.94	48.83	-	0.93–2.3	2.13–5.00	227.5–278.4	2.7–3.2
Rice straw	28.42–48.33	23.22–28.45	12.65–16.72	-	0.86–0.87	2.11–2.25	435–450	24.67–26.33

**Table 2:** The relation of the type of reinforcement and mechanical properties of non-asbestos brake pads.

No.	Type of Reinforcement	Fabrication Method	Weight Fraction (wt.%)	Hardness	Density (g/cm^3^)	Wear Rate	Coefficient of Friction	Ref.
1	Coconut shell powder	Hot Compression (80 °C, 100 KN/cm^2^, 5 min)	2 wt.	21 (Shore D)	2.05			[32]
			4 wt.	70 (Shore D)	2			
			6 wt.	69.7 (Shore D)	1.89			
			8 wt.	68 (Shore D)	1.7			
			10 wt.	58 (Shore D)	1.6			
2	Grounded coconut shell	Hand lay-up	50 wt.	30 (HRF)	2.55	2.56 (10^−6^ g/min)		[35]
			40 wt.	39 (HRF)	2.54	2.1 (10^−6^ g/min)		
			30 wt.	40 (HRF)	2.45	0.5 (10^−6^ g/min)		
			20 wt.	58 (HRF)	2.22	0.25 (10^−6^ g/min)		
			10 wt.	60 (HRF)	2.15	0.5 (10^−6^ g/min)		
3	Palm kernel shell + coconut shell	Compression (16.75 KN/m^2^, 6 h)	25 wt. + 25 wt.	3.3 (kgf/mm^2^)	2.55	0.2193 (g/min)	0.374	[36]
			38 wt. + 13 wt.	3.41 (kgf/mm^2^)	2.6	0.2733 (g/min)	0.383	
			15 wt. + 36 wt.	3 (kgf/mm^2^)	2.78	0.2007 (g/min)	0.362	
4	Candlenut shell powder + coconut shell powder	Compression (15 KN/m^2^, 4 h)	35 wt. + 25 wt.	87 (HR)		5.28 × 10^−5^ g/mm.s		[37]
			30 wt. + 20 wt.	89 (HR)		4.82 × 10^−5^ g/mm.s		
			25 wt. + 15 wt.	92 (HR)		3.67 × 10^−5^ g/mm.s		
5	Wood powder + coconut fiber + cow bone	Compression (2 tons, 1 h)	0 wt. + 40 wt. + 10 wt.	23.9 (HV)			0.47	[38]
			40 wt. + 0 wt. + 10 wt.	35.4 (HV)			0.41	
			20 wt. + 20 wt. + 10 wt.	32.1 (HV)			0.38	
			25 wt. + 25 wt. + 0 wt.	26.5 (HV)			0.44	
6	Coconut shell powder + sugarcane powder	Hand lay-up	21 wt. + 7 wt.			3.55 × 10^−6^ mg/m	0.448	[43]
			14 wt. + 14 wt.			4.13 × 10^−6^ mg/m	0.434	
			7 wt. + 21 wt.			3.87 × 10^−6^ mg/m	0.395	
7	Coconut fiber	Hot Compression (200 °C, 1000 kgf, 20 min)	29 wt	37.14 HRB		0.323 mm^3^/N·mm	0.454	[44]
8	Bamboo fiber	Hot Compression (200 °C, 1000 kgf, 20 min)	20 wt	44.10 HRB		0.242 mm^3^/N·mm	0.46	
9	Palm kernel fiber	Hand lay-up	10 wt.	2.11 HRC		0.000197 mm^3^/N·m		[45]
			20 wt.	2.75 HRC		0.001970 mm^3^/N·m		
			30 wt.	2.84 HRC		0.000390 mm^3^/N·m		
			40 wt.	2.92 HRC		0.000197 mm^3^/N·m		
			50 wt.	2.98 HRC		0.000098 mm3/N·m		
10	Palm kernel fiber + wheat fiber + nile rose fiber	Hand lay-up	5 wt. + 2 wt. + 3 wt.	1.83 HRC		0.00052 mm^3^/N·m		
			10 wt. + 5 wt. + 5 wt.	2.05 HRC		0.00118 mm^3^/N·m		
			15 wt. + 10 wt. + 5 wt.	2.23 HRC		0.00026 mm^3^/N·m		
			20 wt. + 10 wt. + 10 wt.	2.39 HRC		0.00132 mm^3^/N·m		
			25 wt. + 15 wt. + 10 wt.	2.47 HRC		0.00264 mm^3^/N·m		
11	Kenaf fiber	Hot Compression (120 °C, 20 MPa, 30 min)	25 wt.	87 HRB	1.429	3.48 mg/m	0.43	[46]
			20 wt.	86 HRB	1.513	3.89 mg/m	0.41	
			30 wt.	84 HRB	1.639	4.02 mg/m	0.4	
			35 wt.	82 HRB	1.712	4.33 mg/m	0.39	
			40 wt.	88 HRB	2.012	4.65 mg/m	0.38	
			15 wt.	91 HRB	2.392	4.71 mg/m	0.38	
12	Banana fiber + coconut coir + rice husk	Hand lay-up	10 wt. + 5 wt. + 5 wt.	45.6 (Shore D)			0.6	[47]

**Table 3:** Physical and mechanical properties of phenolic resin [70,75,76,77,78,79].

Properties	Value
Specific gravity	1.12–1.16
Flash point (℃)	72.5
Boiling point (℃)	181.8
Melting point (℃)	100–115
Elongation at break (%)	2
Density (g/cm^3^)	1.2–1.4
No tamped volumetric weight (g/dm^3^)	350–550
Tamped volumetric weigh (g/dm^3^)	600–800
Solubility	acetone, ethyl alcohol, ethyl acetate
pH	7–8.5
Tensile strength (MPa)	34.5–62.1
Tensile Modulus (GPa)	2.76–4.8
Thermal-decomposition temperature (℃)	300 (starting)
Total weight losses (%) during the thermal degradation process (room temperature to 800 ℃)	55.2

**Table 4:** The thermal-decomposition temperatures of the binder resins [66,91,92].

The Binder Resins	Thermal Decomposition Temperature (°C)
Aromatic Ring-Modified Phenolic Resin	488.0
Straight Phenolic Resin	418.5–550
Alkyl-Modified Phenolic Resin	461.5
Silicon-Modified Phenolic Resin	378.8
Acrylic 30%-Modified Phenolic Resin	373.9
Cashew Nut Shell Liquid Modified Resin	431
Melamine Resin	408
Alkyl Benzene modified resin	420

## Data Availability

Data are contained within the article.
